# Hepatitis C virus vaccine design: focus on the humoral immune response

**DOI:** 10.1186/s12929-020-00669-4

**Published:** 2020-07-06

**Authors:** Daniel Sepulveda-Crespo, Salvador Resino, Isidoro Martinez

**Affiliations:** Unidad de Infección Viral e Inmunidad, Centro Nacional de Microbiología, Instituto de Salud Carlos III (Campus Majadahonda), Carretera Majadahonda-Pozuelo, Km 2.2;Majadahonda, 28220 Madrid, Spain

**Keywords:** HCV, Antibody, Vaccine, Humoral immune response, Glycoprotein E1, Glycoprotein E2, Virus neutralization

## Abstract

Despite the recent development of safe and highly effective direct-acting antivirals, hepatitis C virus (HCV) infection remains a significant health problem. In 2016, the World Health Organization set out to reduce the rate of new HCV infections by 90% by 2030. Still, global control of the virus does not seem to be achievable in the absence of an effective vaccine. Current approaches to the development of a vaccine against HCV include the production of recombinant proteins, synthetic peptides, DNA vaccines, virus-like particles, and viral vectors expressing various antigens. In this review, we focus on the development of vaccines targeting the humoral immune response against HCV based on the cumulative evidence supporting the important role of neutralizing antibodies in protection against HCV infection. The main targets of HCV-specific neutralizing antibodies are the glycoproteins E1 and E2. Recent advances in the knowledge of HCV glycoprotein structure and their epitopes, as well as the possibility of getting detailed information on the human antibody repertoire generated by the infection, will allow rational structure-based antigen design to target specific germline antibodies. Although obtaining a vaccine capable of inducing sterilizing immunity will be a difficult task, a vaccine that prevents chronic hepatitis C infections, a more realistic goal in the short term, would have a considerable health impact.

## Background

### The hepatitis C virus

Hepatitis C virus (HCV) is an enveloped, positive-sense single-stranded RNA virus that belongs to the *Hepacivirus* genus within the *Flaviviridae* family. Its genome of 9.6 kb is translated into a single large polyprotein, which is processed by cellular and viral proteases into ten mature proteins, comprised of three structural (core, E1, E2) and seven non-structural (NS) proteins (p7, NS2, NS3, NS4A, NS4B, NS5A, and NS5B) [1]. HCV has high genetic diversity with seven main genotypes and more than 60 subtypes, of which genotype 1 is the most prevalent [2]. The difference at the nucleotide level is approximately 30% between genotypes and 15% between subtypes of the same genotype. Additionally, HCV shows enormous genetic diversity within an infected individual, where it exists in the form of quasispecies generated by the high error rate of the HCV polymerase and the elevated replication rate of the virus. These quasispecies can differ by up to 10% in their nucleotide sequence [2–4].

### The natural history of hepatitis C infection

HCV is an important health problem that affects approximately 1% of the global population [5]. Blood transfusions, nosocomial transmission, sharing equipment between injecting drug users (IDU), and tattoos are recognized as common modes of HCV transmission. There is also evidence that HCV may be transmitted sexually among men who have sex with men (MSM) [6]. Following the initial HCV infection, a variable incubation period follows, after which approximately 25% of subjects clear the virus [7]. Fulminant hepatic failure due to acute HCV infection is rare (< 1%), but is a dramatic clinical syndrome with high mortality. The risk of chronic hepatitis C (CHC) infection is high, and around 75% of patients remain HCV RNA positive after acute hepatitis C [7]. According to the World Health Organization (WHO), 71 million people were living with CHC infections worldwide in 2015, and around 2 million new infections occur each year [5, 8]. The long-term natural history of CHC leads, after many years of fibrosis, to liver cirrhosis in approximately 10–20% of patients within 20–30 years. Once cirrhosis is established, decompensated cirrhosis, end-stage liver disease, and hepatocellular carcinoma may develop [9]. Inevitably, terminal liver disease leads to death or the necessity for liver transplantation [9].

### Worldwide elimination of HCV: the need for a prophylactic vaccine

HCV treatment has changed substantially in the last decade with the appearance of direct-acting antivirals (DAAs) [6], which specifically inhibit the function of various NS proteins essential for viral replication, such as the serine protease (NS3/4a) and the RNA-dependent polymerase (NS5b) [10]. After 2014, the second generation of DAAs was available and dramatically increased the cure rate to more than 95% [11]. Moreover, DAA therapy is safer, and its duration is shorter than interferon therapy, the previous standard of care [12]. Following this therapeutic advance, in 2016 the WHO set out to reduce the rate of new HCV infections by 90% by 2030. This initiative involves the scale-up of HCV screening, risk behavior reduction, and unrestricted access to DAA treatment [13]. Based on this strategy, lowering the total number of HCV-positive people worldwide would therefore reduce de novo infections.

However, in the absence of an effective vaccine, there are some limitations to this approach [14, 15]: 1) HCV treatment itself has several unresolved problems. First, between 2 and 5% of HCV-infected patients are not cured of their HCV infection, and DAA therapy can select for resistant variants that limit the effectiveness of the treatment. Second, DAAs are still expensive and inaccessible in most developing countries. 2) Both acute hepatitis C and CHC are largely asymptomatic, and approximately 80% of people infected worldwide are not aware of their infection. Consequently, only 20% of HCV-infected patients are diagnosed, and only 15% of those have been treated [6]. All undiagnosed and untreated patients continue to develop the disease and are potential transmitters of the virus. Reaching treatment rates greater than 60–70% will be problematic, especially in underdeveloped countries. 3) Many subjects infected with HCV and new HCV infections occur in marginalized populations that are difficult to access, such as people who inject drugs (PWIDs), sex workers, MSM, and incarcerated people. These people have limited access to HCV screening and treatment. 4) HCV clearance with DAA therapy does not protect against reinfection. The immunity generated against HCV during CHC is not usually protective, and HCV reinfection after DAA therapy can hamper elimination targets [16]. 5) Finally, the diagnosis of HCV infection is usually performed at advanced stages of liver fibrosis and, although the HCV treatment is successful, severe liver damage is often not completely reversed.

All these reasons make the development of a prophylactic vaccine very likely necessary to control HCV infection worldwide. Effective vaccination strategies at the population level have been the only reliable method to control the transmission of different viral infections by providing herd immunity [17]. Furthermore, in the case of HCV, sterilizing immunity by vaccination would not be necessary to control transmission in high-risk groups. A vaccine reducing viral titers would be sufficient [18]. Furthermore, it has been modeled that a vaccine with only 30% efficacy would have substantial effects on HCV transmission when administered to a high number of high-risk uninfected PWIDs [19–21].

### Current vaccine approaches

Current approaches to the development of a vaccine against HCV include the production of recombinant proteins, synthetic peptides, DNA vaccines, virus-like particles (VLPs) and viral vectors expressing various antigens [22]. These vaccines are aimed to induce either cellular, humoral, or both immune responses [23]. Interestingly, VLPs are emerging as attractive candidates in HCV vaccine design because they can induce high levels of both cellular and humoral immune responses [24–26]. Nevertheless, further studies are required to discover ways to induce long-lasting and highly protective immune responses.

Vaccines inducing T cell-mediated immunity are usually centered on relatively conserved HCV proteins, such as the NS3, NS4, NS5, and core proteins, which are targets of CD8^+^ T cells [27]. Some pre-clinical assays and phase 1 vaccine trials targeting only T-cell responses have been unsuccessful [23, 28]. A promising vaccine based on priming with chimpanzee adenovirus three coding NS proteins (ChAd3-NS) and boosting with modified vaccinia Ankara virus (MVA-NS) was tested in human volunteers. This regimen induced broad HCV-specific memory CD4^+^ and CD8^+^ T cells [29]. Subsequently, this vaccine was tested in a phase 1/2 trial in PWIDs (clinicaltrials.gov identifier NCT01436357), but no protection was shown in patients with CHC infection [30], highlighting the need for a vaccine that induces humoral immune responses along with cell-mediated immunity. Vaccines aimed to induce humoral immune responses are based on the HCV glycoproteins E1, E2, or the E1E2 heterodimer, which are the main targets of protective broad-spectrum neutralizing antibodies (bnAbs). The current vaccines that induce humoral responses against HCV in pre-clinical and clinical trials have been the subject of recent seminal reviews [23, 28], and are summarized in Table 1 [31–52]. Within this approach, the most effective candidate so far is a recombinant E1E2 (rE1E2) purified protein based on the HCV genotype 1a. This vaccine was protective in chimpanzees after homologous challenge [53], and reduced rates of persistence after heterologous challenge [54]. The rE1E2 protein in an oil-in-water emulsion was safe in humans [43], and induced bnAb response [44, 55], although only in three of 16 vaccinated individuals [44].

**Table 1:** Summary of HCV vaccine candidates based on E1/E2 glycoproteins in preclinical or clinical trials

Vaccine / HCV genotype	Target	Cross-genotype neutralization activity using HCVpp and/or HCVcc	Immunized species	Refs
rE2(Δ123) and rE2(Δ123A7) / HCV 1a (H77c)	E2	1a (H77), 2a (J6), 3a (S52), 5a (SA13)	Guinea pigs	[31]
rHCV E1/E2 with admixed sulfated S-lactosylarchaeol (SLA) archaeosome formulation as adjuvant / HCV 1a (H77)	E1E2	N/A	C57BL/6 x BALB/c F1 mice	[32]
HCVp6-MAP. Six peptides (p6) in a multiple antigenic peptide (MAP) derived from conserved epitopes in E1 (1), E2 (2), NS4B (1), NS5A (1) and NS5B (1) / HCV 4a (ED43)	E1, E2, NS4b, NS5a, NS5b	2a (JFH1) and a chimeric 2a/4a (ED43/JFH1)	BALB/c mice	[33]
DNA vaccine encoding sE1E2 into IMX313P (oligomers by fusion with the oligomerization domain of the C4b-binding protein) or sE1 and sE2 as separate immunogens / HCV 1b (HCV-N)	E1, E2, E1E2	1a (H77.20, UKN1A20.8), 1b (ukn1b5.23), 2a (ukn2a1.2, a2.4), 2b (UKN2B1.1, B2.8), 3a (UKN3A1.28, A1.9, A13.6), 4a (UKN4.11.1, 4.21.16), 5 (UKN5.14.4), 6 (UKN6.5.8, 6.5.340)	BALB/c mice	[34]
HCV-like particles bearing core, E1 and E2 from four genotypes / 1a (H77), 1b (BK), 2a (JFH1), and 3a	E1, E2, core	1a (H77), 1b (BK), 2a (JFH1/J6, JFH1), 3a (HIC-109)	BALB/c mice White Landrace pigs	[35, 36]
Chimeric HBV/HCV virus‐like particles bearing three conservative linear epitopes from E1 and E2 and HVR1 mimotope / N/D	E1, E2	1a (JFH1/H77, H77C/JFH1), 1b (Hebei, J4/JFH1), 2a (JFH1/J6, JFH1)	BALB/c mice	[37]
rE2(Δ123) / HCV 1a (H77c)	E2	1a (H77c), 2a (J6), 3a (S52), 4a (ED43), 5a (SA13), 6a (EUHK2), 7a (QC69)	Albino Dunkin Hartley guinea pigs	[38]
rHCV E1/E2 with MF59C.1 as an adjuvant / HCV 1a	E1E2	Genotype 1a/1b patients	Humans (Phase Ib)	[39]
Chimeric HBV/HCV virus‐like particles bearing E1 or E2 / HCV 1a (JFH1/H77)	E1, E2	1a (JFH1/H77; 7a), 1b (JFH1/J4; UKN5.23), 2a (JFH1 WT; UKN 2a1.2), 3 (JFH1/S52; UKN3A.1.28)	New Zealand rabbits	[40]
rHCV E1E2 / HCV 1a (HCV-1)	E1E2	1a (H77), 2a (J6), 3a (S52), 4a (ED43), 5a (SA13), 6a (HK6a)	Chimpanzees	[41]
HCV virus-like particles bearing E1E2 or E1 / HCV 1a (H77)	E1, E1E2	1a (H77), 1b (CG1b, CON1), 2a (JFH-1), 2b (UKN2B), 4c (UKN4)	Macaques (*Macaca fascicularis*) Human CD46 ± IFNαβR-/- mice	[42]
rHCV E1/E2 with MF59C.1 as an adjuvant (oil-in water emulsion) / HCV 1a	E1E2	1a (HCV-1, H77), 1b (UKN1B 12.6), 2a (J6), 3a (S52), 4a (UKN4.11.1), 5a (SA13)	C57BL/6J mice, macaques (*Macaca mulatta*), humans (Phase I, NCT00500747)	[43–46]
DNA vaccine expressing HCV Core, E1 and E2 / HCV 1b (CIGB-230)	E1, E2, core	N/A	Humans (Phase I)	[47–49]
HCV virus‐like particles bearing core, E1, and E2 with AS01B as an adjuvant (a combination of monophosphoryl lipid A and QS21 saponin) / HCV 1b (CG1b)	E1, E2, core	N/A	Chimpanzees (Pan Troglodytes)	[50]
rHCV E1 with aluminum hydroxide as an adjuvant / HCV 1b	E1	N/A	Humans (Phase I)	[51]
DNA vaccine expressing HCV E2 / HCV 1a	E2	N/A	Chimpanzees (Pan Troglodytes)	[52]

**Δ123**: E2‐receptor‐binding domain lacking hypervariable region (HVR) 1 and 2, and the intergenotypic variable region (igVR) (384-408) or replaced with glutathione disulfide linkers (461-485 and570-580); **Δ123A7**: a disulfide-minimized version that contains seven cysteine to alanine mutations (A7: C452A, C486A, C569A, C581A, C585A, C597A, C652A); **HCVcc**: cell-cultured viruses; **HCVpp**: HCV pseudoparticles; **N/A**: Cross-reactive neutralizing antibodies not evaluated; **N/D**: Origin not indicated

In this review, we focus on the development of vaccines targeting the humoral immune response against HCV due to the cumulative evidence supporting the important role of cross-reactive bnAbs in protection against HCV infection. Moreover, recent crucial information about the structure of HCV glycoproteins, their epitopes, and the protective antibody response in humans opens new and exciting expectations in this field. A full description of potential vaccines inducing T cell-mediated immunity and the role of T-cell responses in HCV clearance and protection from reinfection is beyond the scope of the present review. Therefore, we refer the reader to recent excellent seminal reviews for further reading [23, 56].

## The humoral immune response against HCV infection: evidence supporting antibody-based vaccines

### Animal models

There is ample evidence that passive immunization in animal models with HCV-specific nAbs may protect from infection by homologous and heterologous HCV strains and completely clear the acute infection [37, 57–67]. The first in vivo studies demonstrating that nAbs protect against homologous HCV were conducted in chimpanzees. Rabbit hyperimmune serum to the synthetic hypervariable region 1 (HVR1) of HCV E2 glycoprotein, as well as plasma from a CHC patient, neutralized the infectivity of homologous HCV in chimpanzees [59, 60]. The administration of the monoclonal nAb HCV1 (directed against the E2 glycoprotein) prevented the infection of a chimpanzee with HCV genotype 1a, and reduced viral load in acutely and chronically-infected animals [66]. A study conducted by Bukh et al. found a prolonged suppression of HCV replication after challenge with homologous, but not heterologous genotypes, in chimpanzees passively immunized with nAbs from an individual with chronic HCV genotype 1a infection [57]. Protection against homologous challenge was also observed in human liver chimeric mouse models after the infusion of nAbs from CHC patients [65, 67], or a pool of three monoclonal nAbs (AR3A, 3B and 4A) targeting the HCV E2 and E1E2 complex [58].

Due to the high genetic diversity across HCV genomes, the early development of bnAbs capable of blocking infection with multiple heterologous HCV strains is a challenge. Although several studies have shown that active or passive immunization protects against heterologous HCV challenge in chimpanzees [62] and humanized mice [37, 61, 63, 64], this phenomenon is not universal because not all genotypes are blocked.

The use of chimpanzees as a model for the study of humoral immune responses against HCV infection has a great advantage due to its genetic similarity to humans. However, high costs and ethical concerns limit its use [68]. Thus, alternative animal models, particularly humanized mice, are under development to create the ‘ideal’ model fully mimicking clinical settings. Some aspects that can be improved include the humanization levels of hepatocytes and immune cells, the elimination of host-specific factors that block HCV infection, and the humanization of the liver sinusoidal endothelium [69].

### Humans

Beyond animal models, human studies are essential to understand nAbs-mediated humoral immune responses against natural HCV infection and to develop an effective vaccine. In this regard, substantial progress has been made in recent years. HCV-specific nAbs can be detected in the serum of infected people approximately 8–12 weeks after HCV infection [70, 71], although the range is flexible according to the patient’s clinical history [72]. Several studies have shown that nAbs-mediated humoral immune response is long-lasting, necessary to control and clear HCV infection, and can protect from HCV reinfection [22, 28, 46, 73–83]. High-titers and rapid nAb responses have been detected in patients who have spontaneously resolved an acute HCV infection, while a delayed or absent nAb response is associated with HCV persistence and chronicity [75, 78, 79, 82, 84, 85]. NAbs can also be developed during CHC, although it usually takes a long time [86–89]. These nAbs from CHC patients cannot clear the CHC infection spontaneously, likely due to HCV escape mutations at nAb recognition sites [90, 91]. However, this nAb-mediated humoral immunity is associated with reduced liver fibrosis [81]. Interestingly, an exceptional case was observed in a patient with CHC infection who spontaneously cleared the HCV after a strong development of cross-reactive nAbs [80]. BnAbs against HCV were also detected in human immunodeficiency virus (HIV)/HCV-chronic co-infected patients, although they declined or disappeared in many patients after HCV clearance with therapy [92].

Several recent findings have boosted interest in the potential of protective nAbs against HCV, stressing the importance of bnAbs to protect from different HCV genotypes and to limit reinfections [93]. The existence of protective immunity against HCV reinfection with different genotypes remains controversial. Although some studies reported only limited protection against heterologous reinfection [79, 94–96], others showed an apparent cross-genotype immunity [77, 97]. In any case, it is becoming clear that a diverse bnAb response can protect from HCV infections of various genotypes and is associated with spontaneous HCV clearance [78, 79, 98, 99]. In this regard, it has been shown that the combination of distinct human nAbs had complementary and synergistic effects on the neutralization of diverse HCV strains [98, 100, 101]. Additional evidence comes from research in which nAbs targeting multiple epitopes were isolated from people who cleared HCV infection [84, 101], studies showing synergy between nAbs [102], and experiments in a mouse model where HCV infection was eliminated by using a mixture of HCV-specific nAbs [58].

## The envelope glycoproteins E1 and E2

**Fig. 1:**
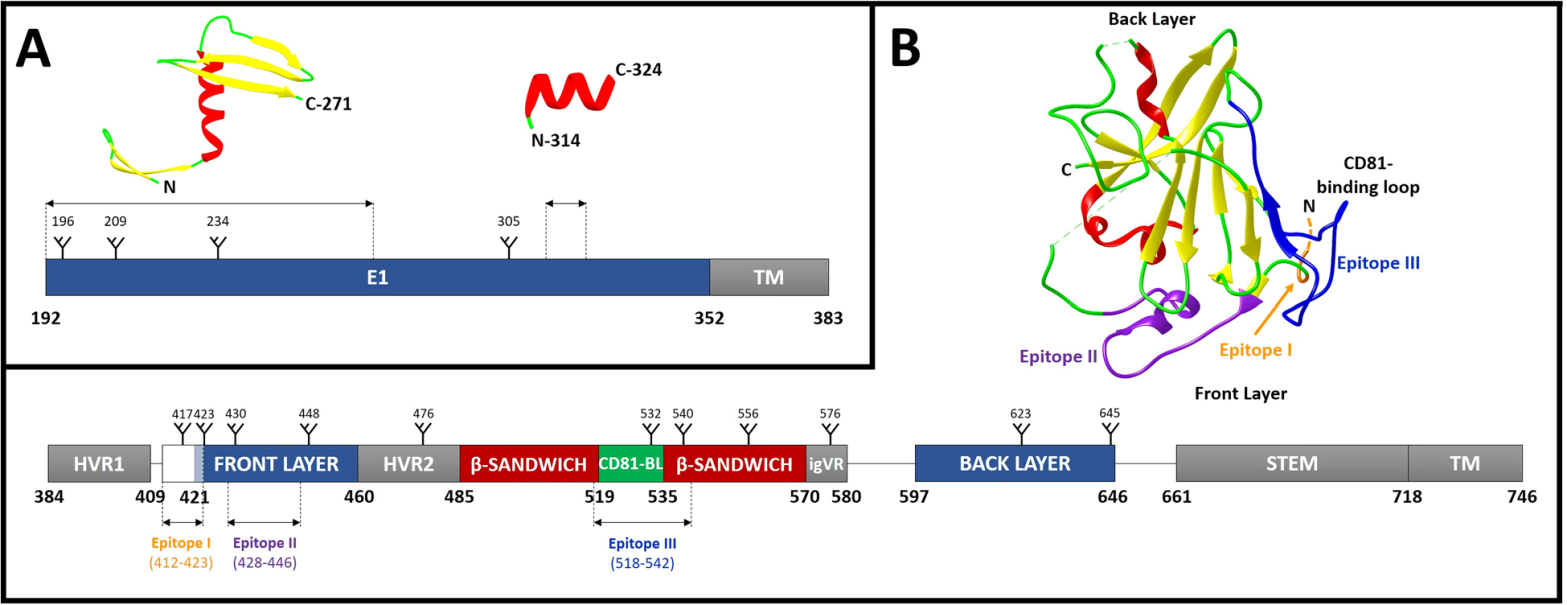
Hepatitis C E1 and E2 glycoprotein structures. **a** Linear diagram of HCV E1 (aa192–383) and crystal structures of E1 segments aa192–271 (PDB: 4UOI) and aa314–324 (PDB: 4N0Y). **b** Linear diagram of HCV E2 (aa384–746) and ribbon representation of the E2 crystal structure (PDB: 4MWF). E2 is divided into the following structural components: three hypervariable regions (HVR1, HVR2 and igVR), a front layer, two β-sandwich regions, CD81 binding loop, a back layer followed by the stem region and transmembrane (TM) domain. The neutralizing face with epitopes I (orange), II (violet) and III (blue) is indicated. **a-b** N-linked glycosylation sites as tree-like representations and well-defined regions containing α-helices and β-sheets are shown in the linear diagram and X-ray crystallographic structure of both glycoproteins, respectively

The main targets of HCV-specific nAbs are the glycoproteins E1 (aa192–383 of the polyprotein) and E2 (aa384–746). They are type-I transmembrane proteins, highly glycosylated, with an N-terminal ectodomain and a C-terminal hydrophobic domain anchoring them to the membrane (Fig. 1). They form E1E2 heterodimers that mediate the entry of HCV into the cell through a complex process involving several receptors and co-receptors, including tetraspanin CD81, the “scavenger” receptor SRB1, and the tight junction membrane proteins claudin 1 and occludin [103]. The virus is internalized by clathrin-dependent endocytosis, and the viral genome is released into the cytoplasm by fusion of the viral membrane with the endosome at low pH, a process also mediated by the E1E2 glycoproteins.

The E1 protein is smaller and less variable than E2. E1 is poorly characterized since only crystal structures for two discrete fragments containing residues 192–271 [104] and 314–324 [105] have been resolved so far (Fig. 1a).

In recent years, two groups have managed to crystallize the ectodomain of E2 together with fragments of two different antibodies, which has constituted a significant advance in the knowledge of the structure of this protein [106, 107]. The two crystal structures show that the E2 ectodomain contains a central immunoglobulin-like β-sandwich highly stabilized by conserved disulfide bonds. This central region is flanked by an N-terminal “front layer” consisting of a β-strand and a short α-helix, and a C-terminal “back layer” containing antiparallel β-sheets and short α-helices [106, 107] (Fig. 1b). However, there are still many questions to be resolved in this field, such as the fact that the crystallized structures differ in the formation of disulfide bridges; important regions of E2 are missing in the structures; E2 was not entirely glycosylated; and, finally, E2 crystallization has been obtained in the absence of E1.

### E1 and E2 epitopes

The elucidation of the E2 structure has led to significant progress in the identification of different antigenic domains and regions of the protein, which will undoubtedly result in the more rational design of vaccines capable of inducing nAbs [22]. E2 is the most variable protein of HCV and the main target of nAbs. Therefore, studies of B-cell based vaccines have focused on this protein. Most of the E2 variability is located in three hypervariable regions: the hypervariable region 1 (HVR1, aa384–409), the hypervariable region 2 (HVR2, aa460–485) and the intergenotypic variable region (igVR, aa570–580) [108]. HVR1 is an immunodominant motif located at the N-terminal end of the protein that mutates during infection, generating escape variants to HCV-specific nAbs.

In contrast, other regions of E2 show moderate variability or are conserved across different genotypes, including areas necessary for the interaction between HCV and cellular receptors, mainly the CD81-binding site. This site is composed of conserved residues from three different regions of E2 that define three epitopes targeted by bnAb [109–111]: Epitope I is located at the N-terminal region (aa412–423); epitope II is at the front layer (aa428–446); and epitope III at the CD81-binding loop (CD81bl) (aa518–542) (Fig. 1b). However, distinct nomenclature is used in different laboratories to describe overlapping antigenic parts of the protein, which may be confusing to the reader. Thus, in addition to the above-mentioned epitopes, five antigenic regions (ARs1–5) [112], and five domains (A-E) [113] have been described. Epitope I shares key residues with domain E; epitope II with domains B, D, and AR3; and epitope III with domain B and AR3 [108, 111]. Furthermore, contact residues on both E1 and E2 are required for some antibodies mediating broad virus neutralization [112]. These residues lie in the AR4 and AR5 regions. In individuals with acute HCV infection, nAb responses to AR3/domain B are dominant [114], and nAbs targeting this region are usually isolated from B lymphocytes of HCV-infected patients [89, 115, 116].

Less is known about the immunogenic regions on E1, but bnAbs have also been described for this protein. The N-terminus (aa192–202) [117] and the fragment encompassing residues 313–328 [105, 118] have been identified as sites inducing nAbs.

## Antibody-based vaccine development

### Challenges for vaccine development: viral strategies to evade antibody neutralization

**Fig. 2:**
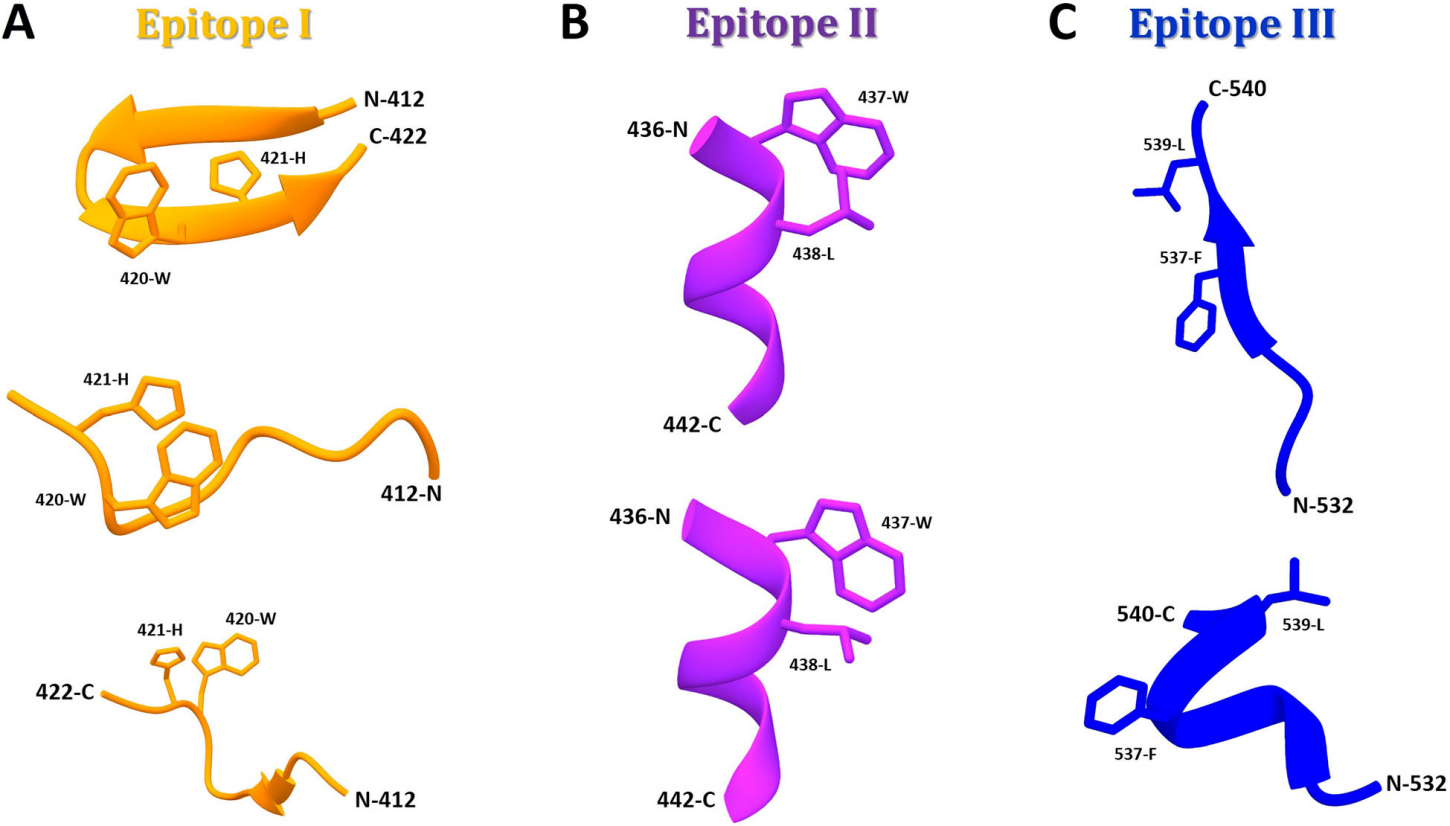
E2 epitopes (I to III) adopt distinct conformations when complexed with different antibodies. **a** Conformations of epitope I: (Upper) Closed β-hairpin in complex with nAb HCV1 (PDB: 4DGY); (Middle) extended-coil conformation in complex with nAb 3/11 (PDB: 4WHY); and (Lower) intermediate-coil conformation with an anti-parallel β-sheet in complex with nAb HC33.1 (PDB:4XVJ). **b** Conformations of epitope II: (Upper) Preferred state of E2 in the viral particle containing a short 1.5-turn α-helix (aa437–442) and an extended conformation (aa443–446, not shown) in complex with nAb HC84.27 (PDB: 4JZO); and (Lower) conformational changes through short α-helix flipping out (‘open state’) to expose aa437(W) and aa438(L) residues for nAb mAb#8 (PDB: 4HZL). **c** Conformations of epitope III: (Upper) Open and stabilized strand in β-sheet conformation with aa537(F) and aa539(L) residues flipped out into the hydrophobic part of the Ig-like domain in complex with AR3C Fab (PDB: 4MWF); and (Lower) helical disordered conformation with aa537(F) and aa539(L) residues solvent-exposed in complex with non-nAb DAO5 (PDB: 5NPJ)

HCV has evolved several mechanisms to counteract antibody neutralization. Firstly, the high mutation rate of HCV promotes the generation of many genetically and antigenically different genotypes, subtypes, and quasispecies [2–4]. Most of the variability is accumulated in the E1 and E2 glycoproteins and contributes to evade the host immune response [119]. As described before, hypervariable regions of E2 are immunodominant and induce isolate-specific or non-nAbs. These hypervariable regions sometimes mask more conserved epitopes, preventing their recognition by nAbs [120, 121]. What is more, some conserved epitopes that participate in receptor recognition show conformational flexibility, which may facilitate escaping from cross-reactive nAbs [122] (Fig. 2). Glycans also contribute to conserved epitope shielding in E2 [123]. E2 contains 11 highly conserved N-linked glycosylation sites, some of which mask the binding site to the cellular CD81 receptor [124–126]. Host-derived lipoproteins, which form part of the mature HCV virion, also hide relevant nAb epitopes [127, 128]. Furthermore, HCV-infected cells in cell culture generate lipid droplets containing the E2 glycoprotein [129]. These droplets may act as antibody decoys, lowering the number of antibodies available to neutralize the virus. Yet another mechanism of HCV to evade antibody recognition is its capacity to spread through direct cell-to-cell transmission [130]. Finally, it has been shown that the enhanced resistance to interferon-induced transmembrane proteins (IFITMs) observed in some HCV variants favors escape from nAbs [131]. IFITMs block viral entry by modulating membrane properties, which improve antibody-mediated neutralization [131].

### Rational immunogen design for antibody-based vaccines

Despite encouraging results, the goal of developing an HCV vaccine remains a challenge. As stated previously, many reasons make it difficult to achieve. However, increasing knowledge about the HCV glycoprotein epitopes offers the opportunity to design immunogens to avoid the induction of isolate-specific or non-nAbs while potentiating the induction of bnAbs. In this regard, an interesting approach has been the generation of an E2 glycoprotein with deleted HVR1, HVR2 and IgVR [132, 133]. This protein elicited bnAbs after immunization of guinea pigs while inducing reduced levels of non-nAbs [38].

N-glycans in E1 and E2 mask epitopes targeted by nAbs [123]. Therefore, the deletion of these glycans may induce a more potent nAb response against HCV. Accordingly, the removal of different N-glycosylation sites both in E1 and E2 improved its immunogenicity and led to increased bnAb responses [134–137]. Interestingly, the glycosylation pattern of E2 can also affect its immunogenicity. Thus, E2 expressed in insect cells showed increased bnAbs as compared to E2 expressed in mammalian cells [64].

In recent years, conformational flexibility of some conserved broadly neutralizing epitopes in HCV E2 has become apparent [110, 138]. For example, the epitope I (AS412) can adopt at least three distinct conformations when complexed with different antibodies: extended [139], β-hairpin [140], and an intermediate conformation [141] (Fig. 2a). Epitopes II (AS434) (Fig. 2b) and III (CD81bl) (Fig. 2c) also display structural flexibility [61, 142–145]. This flexibility appears to be a mechanism to evade nAbs and has important consequences for vaccine design [146]. Thus, future vaccines could require stabilization of neutralizing epitopes, as has been proposed for other viruses such as HIV [147, 148], respiratory syncytial virus (RSV) [149–151], and severe acute respiratory syndrome coronavirus 2 (SARS-CoV-2) [152, 153]. A cyclic variant of the epitope I of E2 stabilized in the β-hairpin conformation was designed first [154]. However, this variant was unable to induce nAbs. Subsequently, another version of the epitope I in the β-hairpin conformation was generated based on the θ-defensin structure [146]. This construct induced nAbs in mice, but the response was still low. Further efforts are required to improve the immunogenicity of structure-based HCV epitopes, including incorporation to virus-like particles or nanoparticles [155].

Another important piece of information that is becoming available is a detailed picture of the human humoral immune response against HCV [86–89]. For example, it is now known that bnAbs in HCV-infected patients are predominantly induced by AR3/domain B and domain C of E2 [155], and specific combinations of these antibodies together with antibodies targeting the E1E2 complex (AR4) are associated with natural HCV clearance [98]. Thus, antigen design focused on eliciting antibodies against these regions should be considered in a potential vaccine. Moreover, the analysis of the interactions of the natural antibody repertoire generated by the infection with the E1E2 glycoproteins will aid the design of new effective vaccines. In this regard, it has been reported recently that potent anti-HCV cross-reactive nAbs with little somatic hypermutations are derived from human *VH1–69* genes [74, 84, 156]. These germline-encoded antibodies are also precursors of a large portion of specific nAbs against other viruses, such as HIV, influenza, and RSV [157–159]. Structural has shown that the cross-neutralizing activity of those antibodies is related to their long complementarity-determining regions (CDR) H3, which contain a disulfide motif that interacts with conserved E2 epitopes [106, 160–162]. Additionally, an ultralong CDRH2 favors extensive contact with E2 [163]. These results underline the potential advantages of producing *VH1–69*-derived nAbs by vaccination.

## Conclusions

**Fig. 3:**
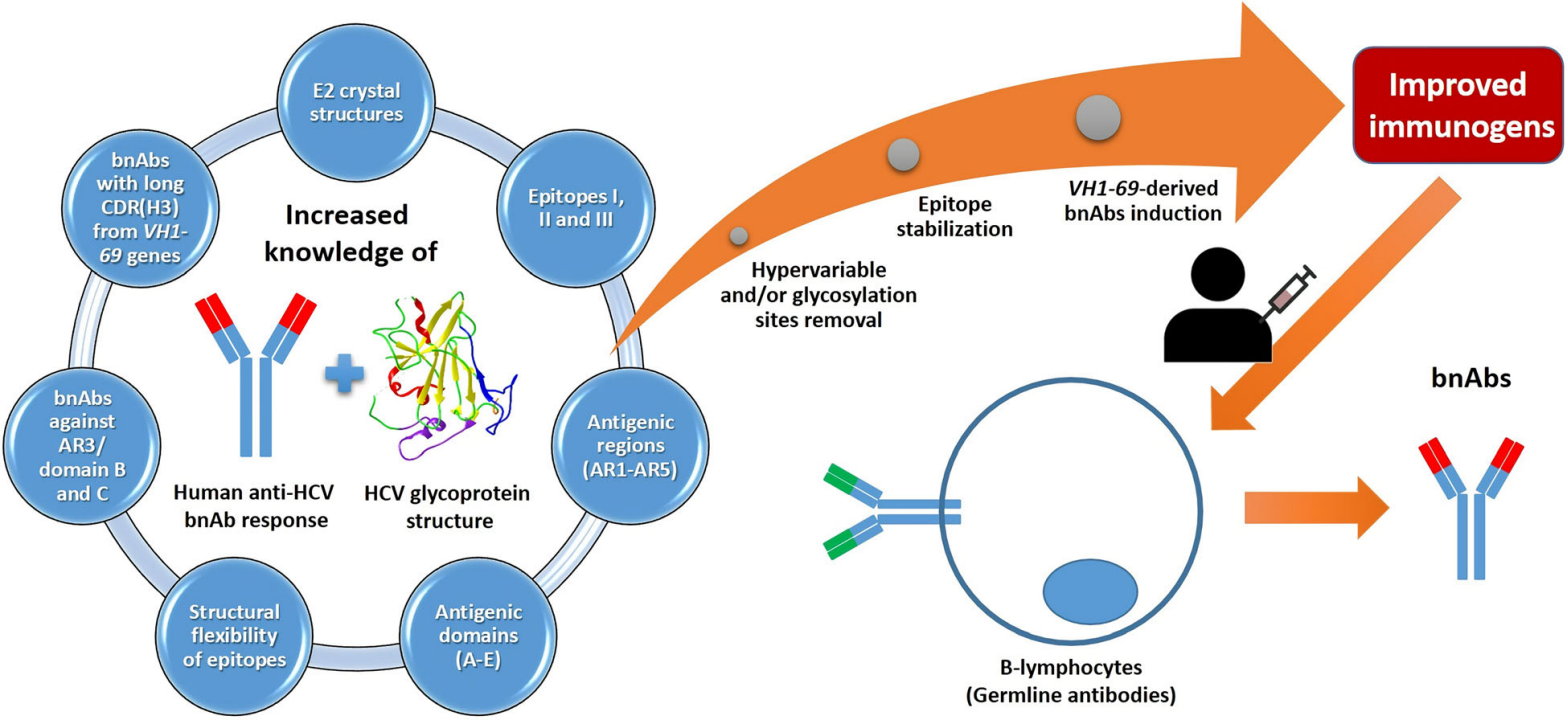
Summary of the key topics discussed in this review

Despite the impressive efficacy of DAA treatment against HCV, it is unlikely that the virus will be controlled entirely without a prophylactic vaccine. Cumulative evidence from animal models and humans strongly indicates that bnAbs can protect from HCV infection. Recent advances in the knowledge of HCV E1 and E2 glycoprotein structure, and the human antibody repertoire generated by HCV infection, will allow rational structure-based antigen design to induce bnAbs (Fig. 3). Achieving sterilizing immunity by vaccination will be a difficult task. However, a vaccine preventing CHC infections, a reasonable goal in the short term, would have a substantial health impact.

## Data Availability

Not applicable.

## Abbreviations

bnAbs: Broad-spectrum neutralizing antibodies
CD81bl: CD81 binding loop
CHC: Chronic hepatitis C
CDR: Complementarity-determining region
DAAs: Direct-acting antivirals
HCV: Hepatitis C virus
HIV: Human immunodeficiency virus
HVR1: Hypervariable region 1
HVR2: Hypervariable region 2
IDU: Injecting drug user
IFITMs: Interferon-induced transmembrane proteins
igVR: Intergenotypic variable region
MSM: Men who have sex with men
MVA: Modified Vaccinia Virus Ankara
nAbs: Neutralizing antibodies
NS: Non-structural
PWIDs: People who inject drugs
RSV: Respiratory syncytial virus
NS5b: RNA-dependent polymerase
NS3/4A: Serine protease
SARS-CoV-2: Severe acute respiratory syndrome coronavirus 2
VLPs: Virus-like particles

## References

[1] Adams RL, Pirakitikulr N, Pyle AM (2017). Functional RNA structures throughout the hepatitis C virus genome. Curr Opin Virol..

[2] Smith DB, Bukh J, Kuiken C, Muerhoff AS, Rice CM, Stapleton JT (2014). Expanded classification of hepatitis C virus into 7 genotypes and 67 subtypes: updated criteria and genotype assignment web resource. Hepatology..

[3] Forns X, Purcell RH, Bukh J (1999). Quasispecies in viral persistence and pathogenesis of hepatitis C virus. Trends Microbiol..

[4] Farci P, Shimoda A, Coiana A, Diaz G, Peddis G, Melpolder JC (2000). The outcome of acute hepatitis C predicted by the evolution of the viral quasispecies. Science..

[5] Polaris Observatory HCVC (2017). Global prevalence and genotype distribution of hepatitis C virus infection in 2015: a modelling study. Lancet Gastroenterol Hepatol..

[6] Spearman CW, Dusheiko GM, Hellard M, Sonderup M (2019). Hepatitis C. Lancet..

[7] Westbrook RH, Dusheiko G (2014). Natural history of hepatitis C. J Hepatol..

[8] World Health Organization (WHO). WHO Fact sheets Hepatitis C. https://www.who.int/en/news-room/fact-sheets/detail/hepatitis-c. Accessed 4 May 2020.

[9] Lingala S, Ghany MG (2015). Natural History of Hepatitis C. Gastroenterol Clin North Am..

[10] Ponziani FR, Mangiola F, Binda C, Zocco MA, Siciliano M, Grieco A (2017). Future of liver disease in the era of direct acting antivirals for the treatment of hepatitis C. World J Hepatol..

[11] European Association for the Study of the Liver (2017). Electronic address eee. EASL recommendations on treatment of hepatitis C 2016. J Hepatol..

[12] European Association for Study of L (2014). EASL clinical practice guidelines: management of hepatitis C virus infection. J Hepatol..

[13] World Health Organization (WHO). Draft global health sector strategies. Viral hepatitis. 2016–2021. http://apps.who.int/gb/ebwha/pdf_files/WHA69/A69_32-en.pdf?ua=1. Accessed 4 May 2020.

[14] Calvaruso V, Petta S, Craxi A (2018). Is global elimination of HCV realistic?. Liver Int..

[15] Cox AL (2015). MEDICINE. Global control of hepatitis C virus. Science..

[16] Roingeard P, Beaumont E. Hepatitis C vaccine: 10 good reasons for continuing. Hepatology. 2020. 10.1002/hep.31182 32060946

[17] Fine P, Eames K, Heymann DL (2011). “Herd immunity”: a rough guide. Clin Infect Dis..

[18] Major M, Gutfraind A, Shekhtman L, Cui Q, Kachko A, Cotler SJ (2018). Modeling of patient virus titers suggests that availability of a vaccine could reduce hepatitis C virus transmission among injecting drug users. Sci Transl Med.

[19] Hahn JA, Wylie D, Dill J, Sanchez MS, Lloyd-Smith JO, Page-Shafer K (2009). Potential impact of vaccination on the hepatitis C virus epidemic in injection drug users. Epidemics..

[20] Scott N, McBryde E, Vickerman P, Martin NK, Stone J, Drummer H (2015). The role of a hepatitis C virus vaccine: modelling the benefits alongside direct-acting antiviral treatments. BMC Med..

[21] Stone J, Martin NK, Hickman M, Hellard M, Scott N, McBryde E (2016). The potential impact of a hepatitis C vaccine for people who inject drugs: is a vaccine needed in the age of direct-acting antivirals?. PLoS One..

[22] Fauvelle C, Colpitts CC, Keck ZY, Pierce BG, Foung SK, Baumert TF (2016). Hepatitis C virus vaccine candidates inducing protective neutralizing antibodies. Expert Rev Vaccines..

[23] Bailey JR, Barnes E, Cox AL (2019). Approaches, Progress, and challenges to hepatitis C vaccine development. Gastroenterology..

[24] Masavuli MG, Wijesundara DK, Torresi J, Gowans EJ, Grubor-Bauk B (2017). Preclinical development and production of virus-like particles as vaccine candidates for hepatitis C. Front Microbiol..

[25] Torresi J (2017). The rationale for a preventative HCV virus-like particle (VLP) vaccine. Front Microbiol..

[26] Collett S, Torresi J, Earnest-Silveira L, Christiansen D, Elbourne A, Ramsland PA (2019). Probing and pressing surfaces of hepatitis C virus-like particles. J Colloid Interface Sci..

[27] Ward S, Lauer G, Isba R, Walker B, Klenerman P (2002). Cellular immune responses against hepatitis C virus: the evidence base 2002. Clin Exp Immunol..

[28] Duncan JD, Urbanowicz RA, Tarr AW, Ball JK (2020). Hepatitis C Virus Vaccine: Challenges and Prospects0. Vaccines (Basel)..

[29] Swadling L, Capone S, Antrobus RD, Brown A, Richardson R, Newell EW (2014). A human vaccine strategy based on chimpanzee adenoviral and MVA vectors that primes, boosts, and sustains functional HCV-specific T cell memory. Sci Transl Med.

[30] National Institute of Allergy and Infectious Diseases (NIAID). Trial Evaluating Experimental Hepatitis C Vaccine Concludes. https://www.niaid.nih.gov/news-events/trial-evaluating-experimental-hepatitis-c-vaccine-concludes. Accessed 4 May 2020.

[31] Center RJ, Boo I, Phu L, McGregor J, Poumbourios P, Drummer HE (2020). Enhancing the antigenicity and immunogenicity of monomeric forms of hepatitis C virus E2 for use as a preventive vaccine. J Biol Chem..

[32] Akache B, Deschatelets L, Harrison BA, Dudani R, Stark FC, Jia Y (2019). Effect of Different Adjuvants on the Longevity and Strength of Humoral and Cellular Immune Responses to the HCV Envelope Glycoproteins. Vaccines (Basel)..

[33] Dawood RM, Moustafa RI, Abdelhafez TH, El-Shenawy R, El-Abd Y, Bader El Din NG (2019). A multiepitope peptide vaccine against HCV stimulates neutralizing humoral and persistent cellular responses in mice. BMC Infect Dis.

[34] Masavuli MG, Wijesundara DK, Underwood A, Christiansen D, Earnest-Silveira L, Bull R (2019). A hepatitis C virus DNA vaccine encoding a secreted, Oligomerized Form of Envelope Proteins Is Highly Immunogenic and Elicits Neutralizing Antibodies in Vaccinated Mice. Front Immunol.

[35] Christiansen D, Earnest-Silveira L, Chua B, Meuleman P, Boo I, Grubor-Bauk B (2018). Immunological responses following administration of a genotype 1a/1b/2/3a quadrivalent HCV VLP vaccine. Sci Rep..

[36] Christiansen D, Earnest-Silveira L, Grubor-Bauk B, Wijesundara DK, Boo I, Ramsland PA (2019). Pre-clinical evaluation of a quadrivalent HCV VLP vaccine in pigs following microneedle delivery. Sci Rep..

[37] Wei S, Lei Y, Yang J, Wang X, Shu F, Wei X (2018). Neutralization effects of antibody elicited by chimeric HBV S antigen viral-like particles presenting HCV neutralization epitopes. Vaccine..

[38] Vietheer PT, Boo I, Gu J, McCaffrey K, Edwards S, Owczarek C (2017). The core domain of hepatitis C virus glycoprotein E2 generates potent cross-neutralizing antibodies in Guinea pigs. Hepatology..

[39] Colombatto P, Brunetto MR, Maina AM, Romagnoli V, Almasio P, Rumi MG (2014). HCV E1E2-MF59 vaccine in chronic hepatitis C patients treated with PEG-IFNalpha2a and ribavirin: a randomized controlled trial. J Viral Hepat..

[40] Beaumont E, Patient R, Hourioux C, Dimier-Poisson I, Roingeard P (2013). Chimeric hepatitis B virus/hepatitis C virus envelope proteins elicit broadly neutralizing antibodies and constitute a potential bivalent prophylactic vaccine. Hepatology..

[41] Meunier JC, Gottwein JM, Houghton M, Russell RS, Emerson SU, Bukh J (2011). Vaccine-induced cross-genotype reactive neutralizing antibodies against hepatitis C virus. J Infect Dis..

[42] Garrone P, Fluckiger AC, Mangeot PE, Gauthier E, Dupeyrot-Lacas P, Mancip J (2011). A prime-boost strategy using virus-like particles pseudotyped for HCV proteins triggers broadly neutralizing antibodies in macaques. Sci Transl Med.

[43] Frey SE, Houghton M, Coates S, Abrignani S, Chien D, Rosa D (2010). Safety and immunogenicity of HCV E1E2 vaccine adjuvanted with MF59 administered to healthy adults. Vaccine..

[44] Law JL, Chen C, Wong J, Hockman D, Santer DM, Frey SE (2013). A hepatitis C virus (HCV) vaccine comprising envelope glycoproteins gpE1/gpE2 derived from a single isolate elicits broad cross-genotype neutralizing antibodies in humans. PLoS One..

[45] Kachko A, Frey SE, Sirota L, Ray R, Wells F, Zubkova I (2015). Antibodies to an interfering epitope in hepatitis C virus E2 can mask vaccine-induced neutralizing activity. Hepatology..

[46] Chen F, Nagy K, Chavez D, Willis S, McBride R, Giang E (2020). Antibody responses to immunization with HCV envelope glycoproteins as a baseline for B-cell-based vaccine development. Gastroenterology..

[47] Alvarez-Lajonchere L, Shoukry NH, Gra B, Amador-Canizares Y, Helle F, Bedard N (2009). Immunogenicity of CIGB-230, a therapeutic DNA vaccine preparation, in HCV-chronically infected individuals in a phase I clinical trial. J Viral Hepat..

[48] Castellanos M, Cinza Z, Dorta Z, Veliz G, Vega H, Lorenzo I (2010). Immunization with a DNA vaccine candidate in chronic hepatitis C patients is safe, well tolerated and does not impair immune response induction after anti-hepatitis B vaccination. J Gene Med..

[49] Amador-Canizares Y, Martinez-Donato G, Alvarez-Lajonchere L, Vasallo C, Dausa M, Aguilar-Noriega D (2014). HCV-specific immune responses induced by CIGB-230 in combination with IFN-alpha plus ribavirin. World J Gastroenterol..

[50] Elmowalid GA, Qiao M, Jeong SH, Borg BB, Baumert TF, Sapp RK (2007). Immunization with hepatitis C virus-like particles results in control of hepatitis C virus infection in chimpanzees. Proc Natl Acad Sci U S A..

[51] Leroux-Roels G, Depla E, Hulstaert F, Tobback L, Dincq S, Desmet J (2004). A candidate vaccine based on the hepatitis C E1 protein: tolerability and immunogenicity in healthy volunteers. Vaccine..

[52] Forns X, Payette PJ, Ma X, Satterfield W, Eder G, Mushahwar IK (2000). Vaccination of chimpanzees with plasmid DNA encoding the hepatitis C virus (HCV) envelope E2 protein modified the infection after challenge with homologous monoclonal HCV. Hepatology..

[53] Choo QL, Kuo G, Ralston R, Weiner A, Chien D, Van Nest G (1994). Vaccination of chimpanzees against infection by the hepatitis C virus. Proc Natl Acad Sci U S A..

[54] Houghton M (2011). Prospects for prophylactic and therapeutic vaccines against the hepatitis C viruses. Immunol Rev..

[55] Wong JA, Bhat R, Hockman D, Logan M, Chen C, Levin A (2014). Recombinant hepatitis C virus envelope glycoprotein vaccine elicits antibodies targeting multiple epitopes on the envelope glycoproteins associated with broad cross-neutralization. J Virol..

[56] Smith S, Honegger JR, Walker C. T-cell immunity against the hepatitis C virus: a persistent research priority in an era of highly effective therapy. Cold Spring Harb Perspect Med. 2020. 10.1101/cshperspect.a036954 PMC7778213 32205413

[57] Bukh J, Engle RE, Faulk K, Wang RY, Farci P, Alter HJ (2015). Immunoglobulin with high-titer in vitro cross-neutralizing hepatitis C virus antibodies passively protects chimpanzees from homologous, but not heterologous. Challenge J Virol..

[58] de Jong YP, Dorner M, Mommersteeg MC, Xiao JW, Balazs AB, Robbins JB (2014). Broadly neutralizing antibodies abrogate established hepatitis C virus infection. Sci Transl Med.

[59] Farci P, Alter HJ, Wong DC, Miller RH, Govindarajan S, Engle R (1994). Prevention of hepatitis C virus infection in chimpanzees after antibody-mediated in vitro neutralization. Proc Natl Acad Sci U S A..

[60] Farci P, Shimoda A, Wong D, Cabezon T, De Gioannis D, Strazzera A (1996). Prevention of hepatitis C virus infection in chimpanzees by hyperimmune serum against the hypervariable region 1 of the envelope 2 protein. Proc Natl Acad Sci U S A..

[61] Keck ZY, Wang Y, Lau P, Lund G, Rangarajan S, Fauvelle C (2016). Affinity maturation of a broadly neutralizing human monoclonal antibody that prevents acute hepatitis C virus infection in mice. Hepatology..

[62] Kong L, Giang E, Robbins JB, Stanfield RL, Burton DR, Wilson IA (2012). Structural basis of hepatitis C virus neutralization by broadly neutralizing antibody HCV1. Proc Natl Acad Sci U S A..

[63] Lacek K, Vercauteren K, Grzyb K, Naddeo M, Verhoye L, Slowikowski MP (2012). Novel human SR-BI antibodies prevent infection and dissemination of HCV in vitro and in humanized mice. J Hepatol..

[64] Li D, von Schaewen M, Wang X, Tao W, Zhang Y, Li L (2016). Altered glycosylation patterns increase immunogenicity of a subunit hepatitis C virus vaccine, inducing neutralizing antibodies which confer protection in mice. J Virol..

[65] Meuleman P, Bukh J, Verhoye L, Farhoudi A, Vanwolleghem T, Wang RY (2011). In vivo evaluation of the cross-genotype neutralizing activity of polyclonal antibodies against hepatitis C virus. Hepatology..

[66] Morin TJ, Broering TJ, Leav BA, Blair BM, Rowley KJ, Boucher EN (2012). Human monoclonal antibody HCV1 effectively prevents and treats HCV infection in chimpanzees. PLoS Pathog..

[67] Vanwolleghem T, Bukh J, Meuleman P, Desombere I, Meunier JC, Alter H (2008). Polyclonal immunoglobulins from a chronic hepatitis C virus patient protect human liver-chimeric mice from infection with a homologous hepatitis C virus strain. Hepatology..

[68] Lanford RE, Walker CM, Lemon SM (2017). The chimpanzee model of viral hepatitis: advances in understanding the immune response and treatment of viral hepatitis. ILAR J..

[69] Yong KSM, Her Z, Chen Q (2019). Humanized Mouse Models for the Study of Hepatitis C and Host Interactions. Cells..

[70] Kinchen VJ, Bailey JR (2018). Defining breadth of hepatitis C virus neutralization. Front Immunol..

[71] Chigbu DI, Loonawat R, Sehgal M, Patel D, Jain P. Hepatitis C Virus Infection: Host(−)Virus Interaction and Mechanisms of Viral Persistence. Cells. 2019;8(4). 10.3390/cells8040376 PMC6523734 31027278

[72] Ishii S, Koziel MJ (2008). Immune responses during acute and chronic infection with hepatitis C virus. Clin Immunol..

[73] Walker CM (2017). Designing an HCV vaccine: a unique convergence of prevention and therapy?. Curr Opin Virol..

[74] Chen F, Tzarum N, Wilson IA, Law M (2019). VH1-69 antiviral broadly neutralizing antibodies: genetics, structures, and relevance to rational vaccine design. Curr Opin Virol..

[75] Dowd KA, Netski DM, Wang XH, Cox AL, Ray SC (2009). Selection pressure from neutralizing antibodies drives sequence evolution during acute infection with hepatitis C virus. Gastroenterology..

[76] Kinchen VJ, Cox AL, Bailey JR (2018). Can broadly neutralizing monoclonal antibodies Lead to a hepatitis C virus vaccine?. Trends Microbiol..

[77] Osburn WO, Fisher BE, Dowd KA, Urban G, Liu L, Ray SC (2010). Spontaneous control of primary hepatitis C virus infection and immunity against persistent reinfection. Gastroenterology..

[78] Osburn WO, Snider AE, Wells BL, Latanich R, Bailey JR, Thomas DL (2014). Clearance of hepatitis C infection is associated with the early appearance of broad neutralizing antibody responses. Hepatology..

[79] Pestka JM, Zeisel MB, Blaser E, Schurmann P, Bartosch B, Cosset FL (2007). Rapid induction of virus-neutralizing antibodies and viral clearance in a single-source outbreak of hepatitis C. Proc Natl Acad Sci U S A..

[80] Raghuraman S, Park H, Osburn WO, Winkelstein E, Edlin BR, Rehermann B (2012). Spontaneous clearance of chronic hepatitis C virus infection is associated with appearance of neutralizing antibodies and reversal of T-cell exhaustion. J Infect Dis..

[81] Swann RE, Cowton VM, Robinson MW, Cole SJ, Barclay ST, Mills PR (2016). Broad anti-hepatitis C virus (HCV) antibody responses are associated with improved clinical disease parameters in chronic HCV infection. J Virol..

[82] Walker MR, Leung P, Eltahla AA, Underwood A, Abayasingam A, Brasher NA (2019). Clearance of hepatitis C virus is associated with early and potent but narrowly-directed, Envelope-specific antibodies. Sci Rep.

[83] Zibert A, Meisel H, Kraas W, Schulz A, Jung G, Roggendorf M (1997). Early antibody response against hypervariable region 1 is associated with acute self-limiting infections of hepatitis C virus. Hepatology..

[84] Bailey JR, Flyak AI, Cohen VJ, Li H, Wasilewski LN, Snider AE (2017). Broadly neutralizing antibodies with few somatic mutations and hepatitis C virus clearance. JCI Insight..

[85] Lavillette D, Morice Y, Germanidis G, Donot P, Soulier A, Pagkalos E (2005). Human serum facilitates hepatitis C virus infection, and neutralizing responses inversely correlate with viral replication kinetics at the acute phase of hepatitis C virus infection. J Virol..

[86] Hadlock KG, Lanford RE, Perkins S, Rowe J, Yang Q, Levy S (2000). Human monoclonal antibodies that inhibit binding of hepatitis C virus E2 protein to CD81 and recognize conserved conformational epitopes. J Virol..

[87] Allander T, Drakenberg K, Beyene A, Rosa D, Abrignani S, Houghton M (2000). Recombinant human monoclonal antibodies against different conformational epitopes of the E2 envelope glycoprotein of hepatitis C virus that inhibit its interaction with CD81. J Gen Virol..

[88] Johansson DX, Voisset C, Tarr AW, Aung M, Ball JK, Dubuisson J (2007). Human combinatorial libraries yield rare antibodies that broadly neutralize hepatitis C virus. Proc Natl Acad Sci U S A..

[89] Law M, Maruyama T, Lewis J, Giang E, Tarr AW, Stamataki Z (2008). Broadly neutralizing antibodies protect against hepatitis C virus quasispecies challenge. Nat Med..

[90] Zeisel MB, Cosset FL, Baumert TF (2008). Host neutralizing responses and pathogenesis of hepatitis C virus infection. Hepatology..

[91] Kelly HR, Urbanski M, Burda S, Zhong P, Konings F, Nanfack J (2005). Neutralizing antibody patterns and viral escape in HIV-1 non-B subtype chronically infected treatment-naive individuals. Hum Antibodies..

[92] Vigon L, Vazquez-Moron S, Berenguer J, Gonzalez-Garcia J, Jimenez-Sousa MA, Guardiola JM (2019). Rapid decrease in titer and breadth of neutralizing anti-HCV antibodies in HIV/HCV-coinfected patients who achieved SVR. Sci Rep..

[93] Tarr AW, Urbanowicz RA, Hamed MR, Albecka A, McClure CP, Brown RJ (2011). Hepatitis C patient-derived glycoproteins exhibit marked differences in susceptibility to serum neutralizing antibodies: genetic subtype defines antigenic but not neutralization serotype. J Virol..

[94] Abdel-Hakeem MS, Bedard N, Murphy D, Bruneau J, Shoukry NH (2014). Signatures of protective memory immune responses during hepatitis C virus reinfection. Gastroenterology..

[95] Islam N, Krajden M, Shoveller J, Gustafson P, Gilbert M, Wong J (2017). Hepatitis C cross-genotype immunity and implications for vaccine development. Sci Rep..

[96] Prince AM, Brotman B, Lee DH, Pfahler W, Tricoche N, Andrus L (2005). Protection against chronic hepatitis C virus infection after rechallenge with homologous, but not heterologous, genotypes in a chimpanzee model. J Infect Dis..

[97] Lanford RE, Guerra B, Chavez D, Bigger C, Brasky KM, Wang XH (2004). Cross-genotype immunity to hepatitis C virus. J Virol..

[98] Kinchen VJ, Massaccesi G, Flyak AI, Mankowski MC, Colbert MD, Osburn WO (2019). Plasma deconvolution identifies broadly neutralizing antibodies associated with hepatitis C virus clearance. J Clin Invest..

[99] Keck ZY, Pierce BG, Lau P, Lu J, Wang Y, Underwood A (2019). Broadly neutralizing antibodies from an individual that naturally cleared multiple hepatitis C virus infections uncover molecular determinants for E2 targeting and vaccine design. PLoS Pathog..

[100] Mankowski MC, Kinchen VJ, Wasilewski LN, Flyak AI, Ray SC, Crowe JE (2018). Synergistic anti-HCV broadly neutralizing human monoclonal antibodies with independent mechanisms. Proc Natl Acad Sci U S A..

[101] Colbert MD, Flyak AI, Ogega CO, Kinchen VJ, Massaccesi G, Hernandez M (2019). Broadly Neutralizing Antibodies Targeting New Sites of Vulnerability in Hepatitis C Virus E1E2. J Virol..

[102] Carlsen TH, Pedersen J, Prentoe JC, Giang E, Keck ZY, Mikkelsen LS (2014). Breadth of neutralization and synergy of clinically relevant human monoclonal antibodies against HCV genotypes 1a, 1b, 2a, 2b, 2c, and 3a. Hepatology..

[103] Lindenbach BD, Rice CM (2013). The ins and outs of hepatitis C virus entry and assembly. Nat Rev Microbiol..

[104] El Omari K, Iourin O, Kadlec J, Sutton G, Harlos K, Grimes JM (2014). Unexpected structure for the N-terminal domain of hepatitis C virus envelope glycoprotein E1. Nat Commun..

[105] Kong L, Kadam RU, Giang E, Ruwona TB, Nieusma T, Culhane JC (2015). Structure of hepatitis C virus envelope glycoprotein E1 antigenic site 314-324 in complex with antibody IGH526. J Mol Biol..

[106] Kong L, Giang E, Nieusma T, Kadam RU, Cogburn KE, Hua Y (2013). Hepatitis C virus E2 envelope glycoprotein core structure. Science..

[107] Khan AG, Whidby J, Miller MT, Scarborough H, Zatorski AV, Cygan A (2014). Structure of the core ectodomain of the hepatitis C virus envelope glycoprotein 2. Nature..

[108] Pierce BG, Keck ZY, Foung SK (2016). Viral evasion and challenges of hepatitis C virus vaccine development. Curr Opin Virol..

[109] Drummer HE (2014). Challenges to the development of vaccines to hepatitis C virus that elicit neutralizing antibodies. Front Microbiol..

[110] Tzarum N, Wilson IA, Law M (2018). The neutralizing face of hepatitis C virus E2 envelope glycoprotein. Front Immunol..

[111] Giang E, Dorner M, Prentoe JC, Dreux M, Evans MJ, Bukh J (2012). Human broadly neutralizing antibodies to the envelope glycoprotein complex of hepatitis C virus. Proc Natl Acad Sci U S A..

[112] Keck Z, Wang W, Wang Y, Lau P, Carlsen TH, Prentoe J (2013). Cooperativity in virus neutralization by human monoclonal antibodies to two adjacent regions located at the amino terminus of hepatitis C virus E2 glycoprotein. J Virol..

[113] Brasher NA, Eltahla AA, Underwood A, Boo I, Rizzetto S, Walker MR (2020). B cell immunodominance in primary hepatitis C virus infection. J Hepatol..

[114] Kinchen VJ, Zahid MN, Flyak AI, Soliman MG, Learn GH, Wang S (2018). Broadly neutralizing antibody mediated clearance of human hepatitis C virus infection. Cell Host Microbe..

[115] Keck ZY, Li TK, Xia J, Gal-Tanamy M, Olson O, Li SH (2008). Definition of a conserved immunodominant domain on hepatitis C virus E2 glycoprotein by neutralizing human monoclonal antibodies. J Virol..

[116] Keck ZY, Sung VM, Perkins S, Rowe J, Paul S, Liang TJ (2004). Human monoclonal antibody to hepatitis C virus E1 glycoprotein that blocks virus attachment and viral infectivity. J Virol..

[117] Meunier JC, Russell RS, Goossens V, Priem S, Walter H, Depla E (2008). Isolation and characterization of broadly neutralizing human monoclonal antibodies to the e1 glycoprotein of hepatitis C virus. J Virol..

[118] Liu L, Fisher BE, Dowd KA, Astemborski J, Cox AL, Ray SC (2010). Acceleration of hepatitis C virus envelope evolution in humans is consistent with progressive humoral immune selection during the transition from acute to chronic infection. J Virol..

[119] Bankwitz D, Steinmann E, Bitzegeio J, Ciesek S, Friesland M, Herrmann E (2010). Hepatitis C virus hypervariable region 1 modulates receptor interactions, conceals the CD81 binding site, and protects conserved neutralizing epitopes. J Virol..

[120] Prentoe J, Jensen TB, Meuleman P, Serre SB, Scheel TK, Leroux-Roels G (2011). Hypervariable region 1 differentially impacts viability of hepatitis C virus strains of genotypes 1 to 6 and impairs virus neutralization. J Virol..

[121] Balasco N, Barone D, Sandomenico A, Ruggiero A, Doti N, Berisio R (2017). Structural versatility of hepatitis C virus proteins: implications for the Design of Novel Anti-HCV intervention strategies. Curr Med Chem..

[122] Lavie M, Hanoulle X, Dubuisson J (2018). Glycan shielding and modulation of hepatitis C virus neutralizing antibodies. Front Immunol..

[123] Falkowska E, Kajumo F, Garcia E, Reinus J, Dragic T (2007). Hepatitis C virus envelope glycoprotein E2 glycans modulate entry, CD81 binding, and neutralization. J Virol..

[124] Helle F, Goffard A, Morel V, Duverlie G, McKeating J, Keck ZY (2007). The neutralizing activity of anti-hepatitis C virus antibodies is modulated by specific glycans on the E2 envelope protein. J Virol..

[125] Pantua H, Diao J, Ultsch M, Hazen M, Mathieu M, McCutcheon K (2013). Glycan shifting on hepatitis C virus (HCV) E2 glycoprotein is a mechanism for escape from broadly neutralizing antibodies. J Mol Biol..

[126] Fauvelle C, Felmlee DJ, Crouchet E, Lee J, Heydmann L, Lefevre M (2016). Apolipoprotein E mediates evasion from hepatitis C virus neutralizing antibodies. Gastroenterology..

[127] Dreux M, Pietschmann T, Granier C, Voisset C, Ricard-Blum S, Mangeot PE (2006). High density lipoprotein inhibits hepatitis C virus-neutralizing antibodies by stimulating cell entry via activation of the scavenger receptor BI. J Biol Chem..

[128] Deng L, Jiang W, Wang X, Merz A, Hiet MS, Chen Y (2019). Syntenin regulates hepatitis C virus sensitivity to neutralizing antibody by promoting E2 secretion through exosomes. J Hepatol..

[129] Brimacombe CL, Grove J, Meredith LW, Hu K, Syder AJ, Flores MV (2011). Neutralizing antibody-resistant hepatitis C virus cell-to-cell transmission. J Virol..

[130] Wrensch F, Ligat G, Heydmann L, Schuster C, Zeisel MB, Pessaux P (2019). Interferon-induced Transmembrane proteins mediate viral evasion in acute and chronic hepatitis C virus infection. Hepatology..

[131] McCaffrey K, Boo I, Poumbourios P, Drummer HE (2007). Expression and characterization of a minimal hepatitis C virus glycoprotein E2 core domain that retains CD81 binding. J Virol..

[132] McCaffrey K, Boo I, Owczarek CM, Hardy MP, Perugini MA, Fabri L (2017). An Optimized Hepatitis C Virus E2 Glycoprotein Core Adopts a Functional Homodimer That Efficiently Blocks Virus Entry. J Virol..

[133] Fournillier A, Wychowski C, Boucreux D, Baumert TF, Meunier JC, Jacobs D (2001). Induction of hepatitis C virus E1 envelope protein-specific immune response can be enhanced by mutation of N-glycosylation sites. J Virol..

[134] Liu M, Chen H, Luo F, Li P, Pan Q, Xia B (2007). Deletion of N-glycosylation sites of hepatitis C virus envelope protein E1 enhances specific cellular and humoral immune responses. Vaccine..

[135] Ren Y, Min YQ, Liu M, Chi L, Zhao P, Zhang XL (2016). N-glycosylation-mutated HCV envelope glycoprotein complex enhances antigen-presenting activity and cellular and neutralizing antibody responses. Biochim Biophys Acta..

[136] Clarke JL, Paruch L, Dobrica MO, Caras I, Tucureanu C, Onu A (2017). Lettuce-produced hepatitis C virus E1E2 heterodimer triggers immune responses in mice and antibody production after oral vaccination. Plant Biotechnol J..

[137] Stroh LJ, Nagarathinam K, Krey T (2018). Conformational flexibility in the CD81-binding site of the hepatitis C virus glycoprotein E2. Front Immunol..

[138] Meola A, Tarr AW, England P, Meredith LW, McClure CP, Foung SK (2015). Structural flexibility of a conserved antigenic region in hepatitis C virus glycoprotein E2 recognized by broadly neutralizing antibodies. J Virol..

[139] Kong L, Lee DE, Kadam RU, Liu T, Giang E, Nieusma T (2016). Structural flexibility at a major conserved antibody target on hepatitis C virus E2 antigen. Proc Natl Acad Sci U S A..

[140] Li Y, Pierce BG, Wang Q, Keck ZY, Fuerst TR, Foung SK (2015). Structural basis for penetration of the glycan shield of hepatitis C virus E2 glycoprotein by a broadly neutralizing human antibody. J Biol Chem..

[141] Deng L, Ma L, Virata-Theimer ML, Zhong L, Yan H, Zhao Z (2014). Discrete conformations of epitope II on the hepatitis C virus E2 protein for antibody-mediated neutralization and nonneutralization. Proc Natl Acad Sci U S A..

[142] Deng L, Zhong L, Struble E, Duan H, Ma L, Harman C (2013). Structural evidence for a bifurcated mode of action in the antibody-mediated neutralization of hepatitis C virus. Proc Natl Acad Sci U S A..

[143] Krey T, Meola A, Keck ZY, Damier-Piolle L, Foung SK, Rey FA (2013). Structural basis of HCV neutralization by human monoclonal antibodies resistant to viral neutralization escape. PLoS Pathog..

[144] Vasiliauskaite I, Owsianka A, England P, Khan AG, Cole S, Bankwitz D (2017). Conformational Flexibility in the Immunoglobulin-Like Domain of the Hepatitis C Virus Glycoprotein E2. MBio..

[145] Pierce BG, Boucher EN, Piepenbrink KH, Ejemel M, Rapp CA, Thomas WD (2017). Structure-Based Design of Hepatitis C Virus Vaccines That Elicit Neutralizing Antibody Responses to a Conserved Epitope. J Virol..

[146] de Taeye SW, Ozorowski G (2015). Torrents de la Pena a, Guttman M, Julien JP, van den Kerkhof TL, et al. immunogenicity of stabilized HIV-1 envelope Trimers with reduced exposure of non-neutralizing epitopes. Cell..

[147] Torrents de la Pena A, Julien JP, de Taeye SW, Garces F, Guttman M, Ozorowski G (2017). Improving the immunogenicity of native-like HIV-1 envelope Trimers by Hyperstabilization. Cell Rep..

[148] Joyce MG, Zhang B, Ou L, Chen M, Chuang GY, Druz A (2016). Iterative structure-based improvement of a fusion-glycoprotein vaccine against RSV. Nat Struct Mol Biol..

[149] McLellan JS, Chen M, Joyce MG, Sastry M, Stewart-Jones GB, Yang Y (2013). Structure-based design of a fusion glycoprotein vaccine for respiratory syncytial virus. Science..

[150] Liang B, Ngwuta JO, Surman S, Kabatova B, Liu X, Lingemann M (2017). Improved Prefusion Stability, Optimized Codon Usage, and Augmented Virion Packaging Enhance the Immunogenicity of Respiratory Syncytial Virus Fusion Protein in a Vectored-Vaccine Candidate. J Virol..

[151] Wrapp D, Wang N, Corbett KS, Goldsmith JA, Hsieh CL, Abiona O (2020). Cryo-EM structure of the 2019-nCoV spike in the prefusion conformation. Science..

[152] Walls AC, Park YJ, Tortorici MA, Wall A, McGuire AT, Veesler D (2020). Structure, function, and antigenicity of the SARS-CoV-2 spike glycoprotein. Cell.

[153] Sandomenico A, Leonardi A, Berisio R, Sanguigno L, Foca G, Foca A (2016). Generation and characterization of monoclonal antibodies against a cyclic variant of hepatitis C virus E2 epitope 412-422. J Virol..

[154] Anasir MI, Poh CL (2019). Structural Vaccinology for viral vaccine design. Front Microbiol..

[155] Skinner NE, Bailey JR (2020). Broadly neutralizing antibodies against hepatitis C virus: location, location, location. J Hepatol..

[156] Olbrich A, Wardemann H, Bohm S, Rother K, Colpitts CC, Wrensch F (2019). Repertoire and neutralizing activity of antibodies against hepatitis C virus E2 peptide in patients with spontaneous resolution of hepatitis C. J Infect Dis..

[157] Smith SA, Burton SL, Kilembe W, Lakhi S, Karita E, Price M (2018). VH1-69 utilizing antibodies are capable of mediating non-neutralizing fc-mediated effector functions against the transmitted/founder gp120. Front Immunol..

[158] Gilman MS, Castellanos CA, Chen M, Ngwuta JO, Goodwin E, Moin SM (2016). Rapid profiling of RSV antibody repertoires from the memory B cells of naturally infected adult donors. Sci Immunol.

[159] Lang S, Xie J, Zhu X, Wu NC, Lerner RA, Wilson IA (2017). Antibody 27F3 broadly targets influenza a group 1 and 2 Hemagglutinins through a further variation in VH1-69 antibody orientation on the HA stem. Cell Rep..

[160] Flyak AI, Ruiz S, Colbert MD, Luong T, Crowe JE, Bailey JR (2018). HCV broadly neutralizing antibodies use a CDRH3 disulfide motif to recognize an E2 glycoprotein site that can be targeted for vaccine design. Cell Host Microbe..

[161] Tzarum N, Giang E, Kong L, He L, Prentoe J, Augestad E (2019). Genetic and structural insights into broad neutralization of hepatitis C virus by human VH1–69 antibodies. Sci Adv.

[162] Yi C, Xia J, He L, Ling Z, Wang X, Yan Y, et al. Junctional and somatic hypermutation-induced CX4C motif is critical for the recognition of a highly conserved epitope on HCV E2 by a human broadly neutralizing antibody. Cell Mol Immunol. 2020:1–11 [published online ahead of print]. 10.1038/s41423-020-0403-1 PMC7222171 32235917

[163] Flyak AI, Ruiz SE, Salas J, Rho S, Bailey JR, Bjorkman PJ (2020). An ultralong CDRH2 in HCV neutralizing antibody demonstrates structural plasticity of antibodies against E2 glycoprotein. Elife..

